# Rewiring the connectome: Evidence and effects

**DOI:** 10.1016/j.neubiorev.2018.03.001

**Published:** 2018-05

**Authors:** Sophie H. Bennett, Alastair J. Kirby, Gerald T. Finnerty

**Affiliations:** Department of Basic and Clinical Neuroscience, Institute of Psychiatry, Psychology and Neuroscience, King’s College London, London SE5 8AF, UK

**Keywords:** Learning, Memory, Behaviour, Cognition, Cortex, Synapse, fMRI, Axon, Neuron, Dendrite, Network, Computational, Neuropsychiatric, Stroke

## Abstract

Neuronal connections form the physical basis for communication in the brain. Recently, there has been much interest in mapping the “connectome” to understand how brain structure gives rise to brain function, and ultimately, to behaviour. These attempts to map the connectome have largely assumed that connections are stable once formed. Recent studies, however, indicate that connections in mammalian brains may undergo rewiring during learning and experience-dependent plasticity. This suggests that the connectome is more dynamic than previously thought. To what extent can neural circuitry be rewired in the healthy adult brain? The connectome has been subdivided into multiple levels of scale, from synapses and microcircuits through to long-range tracts. Here, we examine the evidence for rewiring at each level. We then consider the role played by rewiring during learning. We conclude that harnessing rewiring offers new avenues to treat brain diseases.

## Highlights

• Rewiring is a plasticity mechanism that alters connectivity between neurons.
• Evidence for rewiring has been difficult to obtain.
• New evidence indicates that local circuitry is rewired during learning.
• Harnessing rewiring offers new ways to treat psychiatric and neurological diseases.

## 1 Introduction

Connections between neurons form the physical basis for communication in the brain. The pattern of connections between neurons constrains how information flows through neural circuits, and therefore, how those circuits function. Hence, determining the ‘wiring’ of the brain is an important step in understanding how the brain works and how the brain generates cognition and behaviour (Bota et al., 2015; Oh et al., 2014; Sporns et al., 2005; Swanson and Lichtman, 2016; Vogelstein et al., 2014). This idea has driven initiatives to map the entire connectome of the human brain (Glasser et al., 2016; Sporns et al., 2005), the mouse brain (Oh et al., 2014) and the nervous system of invertebrates, such as the fruit fly, *Drosophila melanogaster* (Eichler et al., 2017; Vogelstein et al., 2014).

A key feature of nervous systems is their ability to learn and form memories. Studies of the cellular basis for such changes have largely concentrated on the modification of synaptic strength through long-term potentiation and long-term depression (Cheetham et al., 2014; Martin et al., 2000; Nabavi et al., 2014; Whitlock et al., 2006). An alternative strategy is to modify the wiring pattern by changing the physical connection between neurons (Barnes and Finnerty, 2010; Chklovskii et al., 2004; Ramón y Cajal, 1894, 1911). The extent to which rewiring occurs in the intact adult brain has been difficult to determine. Recent evidence, however, has shown that rewiring contributes to experience-dependent plasticity and learning (Albieri et al., 2015; Barnes et al., 2015). This suggests that the rewiring of neural circuits may underlie changes in behaviour.

In the early stages of the connectome project, it was thought that synapses could change, but the connections would be invariant once established (Sporns et al., 2005). Evidence for rewiring in the intact adult brain raises the question of how stable the connectome truly is. Here, we consider the extent and effects of rewiring the connectome.

## 2 Wiring, rewiring and network science

The connectome is the entire wiring diagram for the brain. This wiring diagram details the neurons and the connections between them. Each connection consists of the presynaptic axon, the postsynaptic dendrites and synapses between the neurons (Barnes and Finnerty, 2010; Cherniak, 1992; Chklovskii et al., 2004). The brain’s wiring diagram comprises the neurons in the brain and all of the presynaptic axons, postsynaptic dendrites and synapses between those neurons.

Rewiring is a structural change to the brain’s wiring diagram. This could occur in multiple ways ranging from changes in synapses through alterations to whole connections between neurons and on to large-scale modifications of the axonal tracts between brain regions. Two distinct questions arise immediately. Firstly, “to what extent can the wiring of a healthy brain be altered?”. Secondly, “what are the effects of rewiring?”.

The extent of rewiring is a straightforward question about how much the structure of the brain can be changed. An important issue concerns the spatial extent of rewiring: are changes in connectivity restricted to local circuits within a brain region, or can the connectivity between brain regions be altered?

The effects of rewiring are less straightforward. Teasing apart the effect of rewiring is helped by subdividing rewiring into two groups. In one group, rewiring is restricted to formation and elimination of individual synapses at existing connections. As a result, rewiring can alter the number of synapses at the connection (Greenough and Bailey, 1988). The second type of rewiring involves either the formation of entirely new connections between neurons or the complete loss of existing connections (Barnes and Finnerty, 2010; Ramón y Cajal, 1894, 1911). This type of rewiring has the capacity to radically reconfigure neural circuits by incorporating new neurons into a circuit and by expelling neurons from the circuit.

**Fig. 1:**
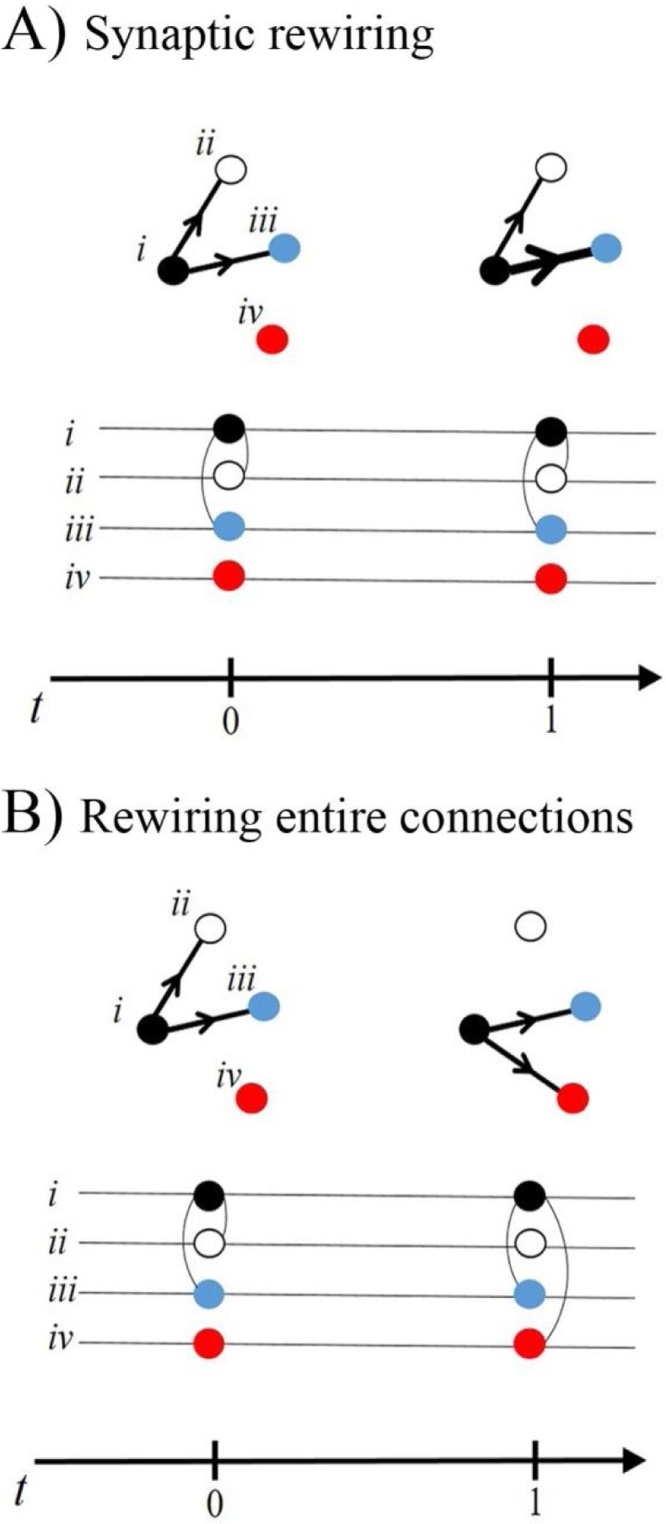
Different effects of rewiring. Each neuron is depicted as a node in a network diagram (Holme and Saramäki, 2012). The connection between a pair of neurons is represented by a line termed and edge, which is drawn between the nodes. Information about the number and strength of synapses forming the connection between neurons can be included in the model by varying the thickness of the edge between the pair of nodes representing the connected neurons. (A) Synaptic rewiring. The thickness of the edge for the i → iii connection increases from *t0* to *t*1 to represent more synapses at this connection. Note that the overall pattern of the edges and the connected nodes in the network do not change. (B) Rewiring of entire connections. The diagram represents loss of the connection, i → ii and formation of a new connection, i → iv. Both the pattern of the edges and the connected nodes in network have changed as a result of this type of rewiring.

The differences between the two groups of rewiring can be seen with highly-reduced models of neural circuits (Fig. 1) (Holme and Saramäki, 2012). Each neuron is shown as a node in a network diagram. The connection between a pair of neurons is represented by a line termed an edge, which is drawn between the nodes (Fig. 1A). Information about the number and strength of synapses forming the connection between neurons can be included in the model by varying the thickness of the edge between the pair of nodes representing the connected neurons. The first type of rewiring, which only involves formation and elimination of individual synapses at existing connections, would be represented in the model by adjusting the thickness of the edges. The nodes in the network are left unchanged (Fig. 1A). In contrast, the second type of rewiring causes more extensive changes in the network diagram (Fig. 1B). Firstly, the edges are affected. The loss of a connection between neurons is modelled by a loss of an edge between the nodes representing the neurons. The formation of a new connection is signified by adding a new edge to link previously unconnected nodes. Secondly, the nodes in the network change as neurons are incorporated into the circuit or expelled from it (Fig. 1B).

Although these models of neural circuits are highly reduced, they capture the different ways that structural rewiring can affect the architecture of a neural circuit. In particular, the models illustrate how loss of whole connections or formation of entirely new connections can change the neurons in a circuit (Fig. 1B). The models that we describe contain minimal functional information about the network. However, the structural changes to the network alone imply that rewiring could dramatically modify the pattern of neural activity in networks.

Network science has used expanded versions of the models we describe to drive further insights into the structure and function connectomes. Initially, graph theory was used to analyse the structure of the connectome. The first neurobiological application was to the connectome of the nematode worm, *C. elegans* (Watts and Strogatz, 1998). Soon afterwards, similar methods were used to analyse the architecture of large-scale brain networks (Bullmore and Sporns, 2009). More recently, network scientists have combined control theory with graph theory to investigate how the wiring in a network supports its function (Braun et al., 2018; Liu et al., 2011). This work led to predictions that have been confirmed experimentally, about which neurons in the *C. elegans* connectome were central to simple motor behaviours elicited by touch (Yan et al., 2017).

Further theoretical work has revealed that networks, which lose established connections and form entirely new connections over time, are more flexible and easier to control (Li et al., 2017). This proposal suggests that rewiring may be a good strategy for changing neural activity in the brain.

In the following sections, we review the evidence that rewiring occurs within the framework of the connectome. We then consider the effects of rewiring and examine the role of rewiring in learning (Table 1). Finally, we ask whether rewiring can be harnessed therapeutically.

**Table 1:** Evidence for rewiring during learning.

Paradigm	Experimental Evidence	Connectome scale	References
Perceptual Learning	Change in dendritic spine density	Micro-	Hofer et al. (2009)
	Reorganization of population activity	Meso-	Chen et al. (2013), Chen et al. (2015), Poort et al. (2015), Yan et al. (2014)

Motor Learning	Increased spine turnover	Micro-	Xu et al. (2009), Yang et al. (2009, 2014)
	Reorganization of population activity	Meso-	Peters et al. (2014)
	Increased grey matter volume	Macro-	Boyke et al. (2008), Draganski et al. (2004)
	Changes in white matter density	Macro-	Scholz et al. (2009)
	New long-range connections	Macro-	Hihara et al. (2006)
	Modular decomposition of functional connectivity	Macro-	Bassett et al. (2011, 2015)

Sensory experience	Increased spine turnover	Micro-	Holtmaat et al. (2006), Trachtenberg et al. (2002), Yang et al. (2009), Chen et al. (2012)
	Increased inhibitory synapse turnover	Micro-	Chen et al. (2012), van Versendaal et al. (2012)
	Formation and elimination of whole neuronal connections	Meso-	Albieri et al. (2015)
	Reorganization of population activity	Meso-	Margolis et al. (2012), Rose et al. (2016), Holtmaat et al. (2006), Trachtenberg et al. (2002)
	BOLD fMRI cortical reorganisation	Macro-	Albieri et al. (2015)
	Axonal sprouting of long-range terminals	Macro-	Oberlaender et al. (2012), Knudsen (1998)

Associative learning	Increase in spine density during passive avoidance learning	Micro-	O’Malley et al. (1998)
	Reorganisation of population activity during associative fear learning	Meso-	Gdalyahu et al. (2012), Grewe et al. (2017)
	Changes in effective connectivity	Macro-	Buchel et al. (1999)
	Axonal sprouting of long-range terminals	Macro-	Boele et al. (2013), Kleim et al. (2002)

Spatial learning	Increase in spine density after motor learning task	Micro-	O’Malley et al. (2000), Moser et al. (1994)
	Increased grey matter volume	Macro-	Maguire et al. (2000)

Rule learning	Changes in effective connectivity	Macro-	Fletcher et al. (1999)

Song learning	Increased spine turnover	Micro-	Roberts et al. (2010)

Adult neurogenesis	Adult-born neurons form new connections with established neurons.	Micro-	Faulkner et al. (2008), Toni et al. (2008)
	Adult-born neurons participate in hippocampal dependant memory.	Meso-	Sahay et al. (2011), Shors et al. (2001)

This table is a non-exhaustive list of publications that have studied rewiring in different learning paradigms.

## 3 Does the connectome rewire?

In this section, we review the evidence for rewiring of the connectome. Addressing this issue requires study of brain architecture at all scales of the connectome.

### 3.1 Macro-connectome, meso-connectome and micro-connectome

**Fig. 2:**
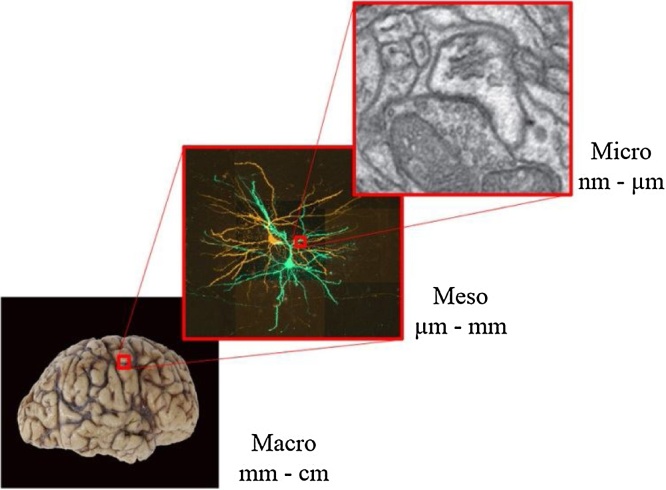
Scales of the connectome. Macro-connectome: Post-mortem human brain. Meso-connectome: Z-stack reconstruction of synaptically-connected pyramidal neurons imaged with confocal microscopy (Cheetham et al., 2007). Micro-connectome: electron microscope section through an asymmetric, presumably excitatory synapse (Cheetham et al., 2014).

Compiling the connectome represents a huge task. Therefore, brain wiring has been described at different scales (Fig. 2) (Sporns et al., 2005; Swanson and Lichtman, 2016). The macro-connectome refers to the distribution of long-range tracts between brain regions. Here, brain region is defined cytoarchitectonically, e.g. using Brodmann areas in humans, or according to animal brain atlases, such as the Allen Brain Atlas for mice (http://mouse.brain-map.org/). An alternative, more recent classification has divided the human cerebral cortex into regions based on cortical architecture, function and connectivity (Glasser et al., 2016). The meso-connectome describes the connections between neurons that compose a circuit within a brain region. Lastly, the micro-connectome characterises connections at the level of individual synapses.

The vast majority of the studies that provide cellular level resolution have been based on invasive techniques and have been performed in rodents, predominantly rats and mice. In contrast, much of the data from non-invasive neuroimaging experiments has been obtained from human studies.

### 3.2 Micro-connectome rewiring

**Fig. 3:**
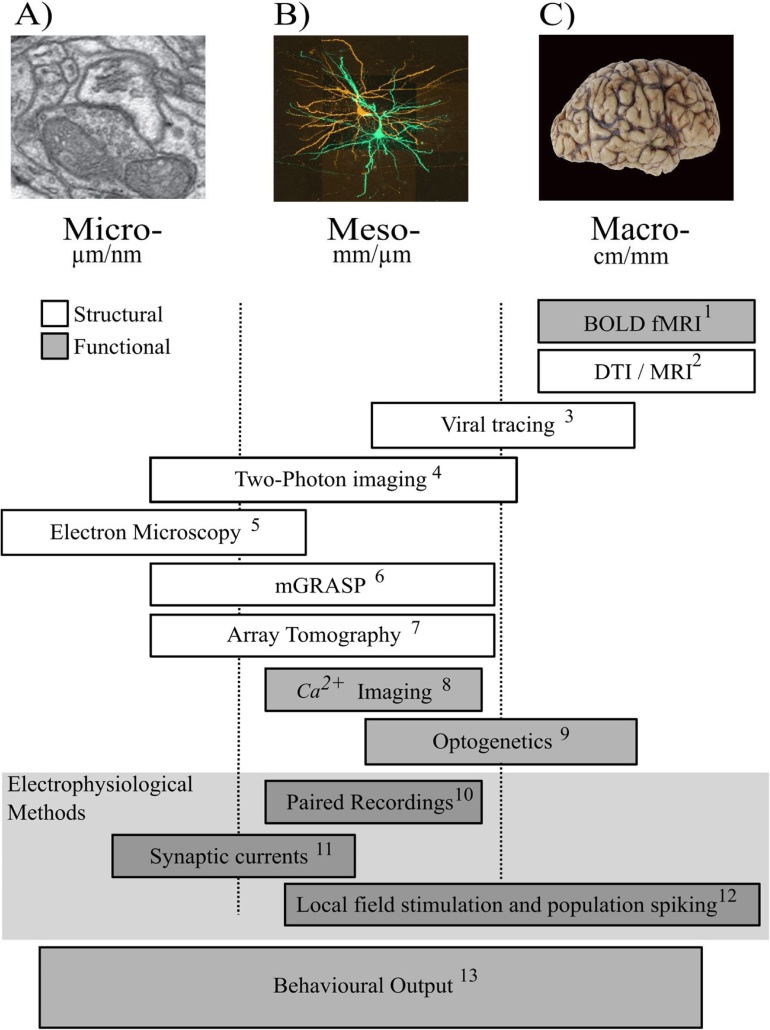
Investigating the connectome. Examples of techniques used to investigate the connectome at different scales. (A) Micro-connectome: electron microscope section through an asymmetric, presumably excitatory synapse (Cheetham et al., 2014). (B) Meso-connectome: Z-stack reconstruction of synaptically-connected pyramidal neurons imaged with confocal microscopy (Cheetham et al., 2007). (C) Human post-mortem brain. (**1**) Functional connectivity is inferred from brain regions exhibiting temporally-correlated fluctuations in blood-oxygen-level-dependent (BOLD) functional MRI signal (Biswal et al., 1995). (**2**) White matter tracts can be studied with diffusion tensor imaging (DTI) tractography (Basser et al., 2000). (**3**) Tracing methods based on molecular tracers or viruses encoding a fluorophore show the long-range axonal projections between brain regions. These tracing studies do not identify both the presynaptic and postsynaptic neurons that form each connection. Transsynaptic tracing technologies have been developed to achieve this e.g. by modifying viruses, such as the rabies virus (DeNardo et al., 2015; Miyamichi et al., 2011; Wickersham et al., 2007; Zingg et al., 2017, 2014). More recently, viral vectors used for tracing have been engineered to encode a fluorophore for tracing and a second protein, such as the calcium indicator GCaMP6s. This facilitates study of the structure of neurons whose activity has been studied *in vivo* (Wertz et al., 2015). (**4**) High-resolution two-photon imaging of neurons filled with a fluorophore has been used to follow structural changes in dendritic spines and axonal boutons during learning and experience-dependent plasticity (Holtmaat and Svoboda, 2009). (**5**) The gold standard for the structural study of synapses is electron microscopy (EM) (Bock et al., 2011; Knott et al., 2002). Focused Ion Beam scanning electron microscopy (FIBSEM) gives serial-section EM images through tissue several micrometres thick (Helmstaedter et al., 2013; Knott et al., 2011). (**6**) mGRASP (mammalian green fluorescent protein (GFP) reconstitution across synaptic partners) combines tracing with the identification of pre-synaptic and post-synaptic structures. It has been used to study excitatory inputs from CA3 onto CA1 pyramidal neurons in the hippocampus (Druckmann et al., 2014). (**7**) Array tomography combines ultrathin sectioning with fluorescent labelling of synaptic proteins (Micheva and Smith, 2007). It has been proposed that combining mGRASP with array tomography would facilitate investigation of the meso-connectome with synaptic level resolution (Rah et al., 2015). (**8**) Calcium imaging uses a calcium-sensitive fluorophore inside the neuron to report when the neuron fires an action potential e.g. (Margolis et al., 2012). (**9**) Optogenetics can be combined with a variety of techniques to investigate functional circuitry at all three scales of the connectome (Kim et al., 2017). (**10–12**) Electrophysiological methods are used to investigate the neural activity in the macro-, meso- and micro-connectome. (**13**) Changes in neural activity within the macro-, meso- or micro-connectome scale circuit may have consequences for behaviour.

The micro-connectome describes the connectome at the level of individual synapses (Bock et al., 2011; Helmstaedter et al., 2013; Kasthuri et al., 2015; Lee et al., 2016; Sporns et al., 2005) (Fig. 3A). Micro-connectome rewiring involves changing either the number of synapses forming a connection between neurons or the dendritic location of those synapses (Barnes and Finnerty, 2010). This is achieved by forming new synapses and eliminating existing synapses. Initial evidence for synaptic rewiring came from histological studies of spine density following new sensory experience or training. The results were mixed. Some studies reported a persistent increase in synapse number (Greenough and Bailey, 1988) whereas other groups detected a transient change (Knott et al., 2002) or found no change in the number of excitatory synapses (Geinisman, 2000). A limitation of these studies is that they only measure total synapse number. It is possible, however, for the distribution of synapses and connectivity to be altered without affecting total synapse number. This would be the case, for example, if the formation of new synapses is offset by the elimination of existing synapses (Cheetham et al., 2007; Barnes and Finnerty, 2010).

The development of *in vivo* two-photon imaging has been a major advance for understanding how the micro-connectome rewires (Holtmaat and Svoboda, 2009) (Fig. 3A). Two-photon imaging allows fluorescent neurons to be imaged repeatedly over weeks. Typically, only the postsynaptic soma and dendritic tree or the presynaptic axon is imaged. The existence of an excitatory synapse is inferred from the presence of a postsynaptic dendritic spine (Knott et al., 2006) or a presynaptic axonal varicosity of an excitatory neuron (De Paola et al., 2006; Grillo et al., 2013; Marik et al., 2010). *in vivo* imaging of inhibitory synapses is more complex, as most inhibitory synapses are formed on the dendritic shaft or soma of the postsynaptic neuron. Therefore, unlike excitatory synapses, the presence of an inhibitory synapse can’t be inferred from a structural marker, such as a dendritic spine. Instead, longitudinal two-photon imaging is achieved by colocalizing two fluorescent markers. One fluorophore labels the postsynaptic neuron, whilst the second fluorophore tags gephyrin, a postsynaptic scaffolding protein at inhibitory synapses (Chen et al., 2012; van Versendaal et al., 2012).

Longitudinal two-photon imaging has revealed that dendritic spines are formed and eliminated on the dendrites of pyramidal neurons (Hofer et al., 2009; Holtmaat et al., 2006; Trachtenberg et al., 2002; Zuo et al., 2005). This spine turnover occurs physiologically in the healthy adult brain. Electron microscopy of the new spines indicates that they form excitatory synapses within days (Knott et al., 2006). Dendritic spine turnover, and presumably excitatory synapse turnover, is increased by sensory experience (Holtmaat et al., 2006; Trachtenberg et al., 2002) and by learning (Xu et al., 2009; Yang et al., 2009). The time course of the increased spine turnover has been studied with motor learning paradigms. Training on a seed reaching task leads to increased spine formation on the apical tufts of layer 5 pyramidal neurons in primary motor cortex within hours (Xu et al., 2009). The new spines have been reported to cluster together on dendritic branches during learning (Fu et al., 2012; Yang et al., 2014) and experience-dependent plasticity (Chen et al., 2012). Increased elimination of dendritic spines occurs after two days so that the spine number is kept in balance (Xu et al., 2009). This finding suggests that rewiring is tightly controlled to keep total synapse number kept relatively constant in the brain.

Rewiring has also been observed at inhibitory synapses. Inhibitory synapses turnover during monocular deprivation (Chen et al., 2012; van Versendaal et al., 2012). Inhibitory synapse rewiring is also observed when sensory input is increased. This has been demonstrated with electron microscopy, which revealed a transient increase in inhibitory synapses onto layer 4 neurons in primary somatosensory cortex following repetitive stimulation of rodents’ whiskers (Knott et al., 2002).

In summary, there is good evidence that the micro-connectome rewires. Excitatory and inhibitory synapses are formed and eliminated physiologically. This process occurs during learning and is increased by sensory experience.

### 3.3 Meso-connectome rewiring

The mesoscale connectome describes the connections between networks of neurons that form local circuits (Fig. 3B). The mesoscale is less concerned with the individual synapses that form connections, but rather focuses on the presence or absence of entire connections between neurons. Rewiring affects mesoscale connectivity if the rewiring changes entire connections between neurons in local circuits. As a result, mesoscale rewiring either incorporates new neurons into a circuit or expels neurons from a circuit (Fig. 1B).

What is the evidence then that mesoscale rewiring occurs? Most detailed experimental studies of synaptic rewiring described in the preceding section (micro-connectome rewiring) are not well suited to study mesoscale rewiring. This is because the pre or post-synaptic partner of the spine or bouton remains unknown. Therefore, it is not clear whether new synapses are formed between neurons that have an existing connection or whether the new synapses create an entirely new connection, and alter mesoscale connectivity.

Detecting changes in mesoscale connectivity requires knowledge of neuron-to-neuron connectivity. However, it is extremely difficult with current techniques to fully resolve neural connectivity at the circuit level unless the connected neurons are within a few hundred micrometres of each other (Albieri et al., 2015; Cheetham et al., 2007; Cossell et al., 2015; Ko et al., 2013, 2011). Longer-range inputs have been studied with a combination of anterograde and retrograde tracers (Zingg et al., 2014) or a viral anterograde tracer combined with serial two-photon tomography to map the axons projecting from the injection site (Oh et al., 2014) (Fig. 3B). These techniques have yielded important information about axonal projections between brain regions. However, these techniques have not been used to examine rewiring.

The occurrence of mesoscale rewiring may be signalled by changes in the neurons that fire in neural circuits. In the neocortex, this can be examined by implanting arrays of microelectrodes or with two-photon calcium imaging (Fig. 3B). Analysis of the firing of multiple neurons attempts to infer connectivity between neurons or the structure of the underlying neural circuits (Aertsen et al., 1989; Doiron et al., 2016; Litwin-Kumar and Doiron, 2014; Moore et al., 1970). Two-photon calcium imaging uses a calcium indicator inside the neuron to report when the neuron fires spikes. The studies give similar overall results. There is reorganization of the neurons that fire action potentials during experience-dependent plasticity (Margolis et al., 2012; Rose et al., 2016), motor learning (Peters et al., 2014), perceptual learning (Chen et al., 2013, 2015; Poort et al., 2015; Yan et al., 2014) and associative learning (Gdalyahu et al., 2012; Grewe et al., 2017). These findings are consistent with rewiring, but may be due to other forms of plasticity.

Direct evidence of rewiring of entire connections comes from studies of local cortical circuits. Electrophysiological recording of synaptically-connected pairs of neurons gives detailed information on both local connectivity and the function of connections (Fig. 3B). Rewiring of cortical microcircuits can be induced by experience-dependent plasticity. Two photon calcium imaging *in vivo* has been combined with electrophysiological recording from the 2-photon imaged neurons *in vitro* to study the neuronal connections of imaged neurons directly. These experiments suggest that rewiring of cortical circuits contributes to the reorganization of neural firing during developmental experience-dependent plasticity in visual cortex (Ko et al., 2013). In mature rodents, altering whisker sensory experience results in a marked increase in local excitatory connections between pyramidal neurons in primary somatosensory cortex, which is followed by a loss of local excitatory connections (Albieri et al., 2015). Collectively, these findings suggest that local circuit rewiring reconfigures the neural circuits in sensory cortex at the meso-connectome level.

It is less clear whether there is rewiring of inhibitory circuitry at the meso-connectome level. This is because few studies have investigated the behaviour of entire inhibitory connections. Electrophysiological recordings of inhibitory circuitry indicate that neither the connectivity nor the functional properties of inhibitory connections involving fast-spiking interneurons change in somatosensory cortex after experience-dependent plasticity (Albieri et al., 2015). In the neocortex, however, interneurons connect with the majority of neighbouring excitatory pyramidal neurons (Albieri et al., 2015). Hence, it may not be necessary to reconfigure inhibitory circuitry by forming entirely new connections and losing established connections. Instead, it is quite plausible that inhibitory control of cortical circuits only requires a change in the number of strength of synapses at existing connections in inhibitory circuits (Fig. 1A).

Incorporation of adult-born neurons into local circuits represents a special case of mesoscale rewiring. Adult-born neurons are generated in the hippocampus and olfactory bulb via neurogenesis. New neuronal connections are first formed by established neurons onto adult-born neurons (Esposito et al., 2005; Ge et al., 2006; Laplagne et al., 2006; Toni et al., 2007). Soon afterwards, adult-born neurons form new connections with established neurons (Faulkner et al., 2008; Toni et al., 2008). These new connections are refined by experience-dependent plasticity (Bergami et al., 2015). The connections involving adult-born neurons are integrated into the existing neural circuitry. This results in reorganization of existing circuitry as new connections compete with established connections (Yasuda et al., 2011). Hence, the evidence suggests that adult-born neurons become fully integrated into mature neural circuits.

Taken together, the experimental evidence suggests that local excitatory circuits rewire during experience-dependent plasticity and hippocampus-dependent learning.

### 3.4 Macro-connectome rewiring

The long-range connections between different brain regions constitute the ‘macroscale’ connectome (Fig. 3C). Rewiring of the macro-connectome occurs if the connections between brain regions are altered. In principle, sprouting of long-range axons could wire up brain regions that were previously unconnected. Similarly, retraction of long-range axons could disconnect brain regions. Less dramatic rewiring of the macro-connectome could occur by keeping the connection between two brain regions, but changing the neurons targeted by the long-range axons. This only requires structural remodelling of the terminal arbor of the long-range axons.

Developments in magnetic resonance imaging (MRI) have dramatically expanded the ways that the macro-connectome of the living brain can be studied. The axons forming the physical connections between brain regions are arranged as either tightly bundled myelinated tracts or more diffuse axon fascicles. These long-distance connections are commonly visualised with tractography based on diffusion tensor imaging (DTI) (Basser et al., 2000; Catani et al., 2012) (Fig. 3C). DTI can have difficulties following axonal fibres in regions of fibre crossing or when fibre direction changes markedly (Jbabdi et al., 2015). Furthermore, it is not clear whether changes in the DTI signal reflect altered myelin thickness (Zikopoulos and Barbas, 2010) or altered density of connections. Despite these issues, DTI has been highly useful in studying white matter tracts in the brain. The structure of the cortex has been studied with volumetric MRI (Lerch et al., 2017).

Using these techniques, macroscale structural changes have been observed during learning. For example, a number of studies report localised increases in grey matter after training (Boyke et al., 2008; Draganski et al., 2004; Maguire et al., 2000). Age-dependent changes in white matter have also been observed. White matter structure is altered by learning during the first two decades of human life, when the white matter tracts are developing (Johansen-Berg et al., 2010). In adults, white matter changes are less marked and are restricted to the region neighbouring the cortex activated during learning (Scholz et al., 2009). In general, there is little evidence that large-scale restructuring of axons and white matter tracts occur in healthy adult brains.

The cellular mechanisms mediating the changes in grey and white matter are unclear. An increase in grey matter volume could be attributable to synaptogenesis and indicate rewiring. However, there are multiple other explanations, such as changes in synapse morphology, gliogenesis, and vascular changes (Zatorre et al., 2012). Likewise, altered white matter structure could reflect changes in axon density, axon diameter, or altered myelination. Change in axon diameter and myelination represent changes to the structure of axons rather than rewiring of axons. Changes in axon density could be due to multiple causes including altered myelination, a change in the extracellular space, or axonal rewiring. A combined fMRI and immmunohistochemical study suggested that the cause of the altered white matter adjacent to “trained” neocortex was increased myelination rather than rewiring (Sampaio-Baptista et al., 2013). In summary, neuroimaging studies indicate that learning is associated with structural changes to grey and white matter, but there is no clear evidence that those changes are due to rewiring.

More direct evidence for rewiring of the macro-connectome has been sought with anatomical tracing studies in animals (Fig. 3C). Tracing methods use molecular tracers or viruses encoding a fluorophore to study long-range connections to and from brain regions. One viral tracing study has presented evidence for macroscale rewiring. In that study, monkeys trained to use tools formed new axonal connections between higher visual areas and the interparietal cortex (Hihara et al., 2006). However, evidence of this kind is rare. An alternative strategy is to change the neuronal targets of long-range connections within local circuitry. Tracing studies suggest that both experience (Knudsen, 1998; Oberlaender et al., 2012) and associative learning (Boele et al., 2013; Kleim et al., 2002) induce rewiring of the macro-connectome in healthy adult brains of animals. The rewiring occurs at the terminal branches of the long-distance axons (Boele et al., 2013; Oberlaender et al., 2012). Hence, the evidence suggests that modification to long-range connections is most likely confined to rewiring of the terminal branches.

In conclusion, there is strong evidence that individual synapses undergo rewiring and that the microconnectome is dynamic. It is also becoming clearer that local “mesoscale” circuitry can be altered during learning. In contrast, long-range connections between brain regions are unlikely to undergo extensive rewiring in the healthy adult brain. Instead, rewiring of long-range connections predominantly affects the terminal arbours of the projecting axons and can, therefore, alter the neurons receiving the axonal inputs.

## 4 Functional connectomics

The definition that we have given of the connectome is concerned with the physical connections between neurons. This definition emphasizes structure and was the original meaning of “connectome”. However, the use of the term connectome in neuroscience has broadened to incorporate functional networks, typically at the level of the macro-connectome. This field has been termed functional connectomics. The functional networks arise from studies of how brain activity varies across different brain regions. The brain activity can be measured with neuroimaging or electrophysiological methods, such as functional magnetic resonance imaging (fMRI), electroencephalography (EEG) or magnetoencephalography (MEG) (Brookes et al., 2011; Matthews and Hampshire, 2016) (Fig. 3C).

As with structural connectomes, functional networks can be represented and analysed with metrics from network science. A key goal has been to probe the connectivity between brain regions. Connectivity has been subdivided into functional connectivity and effective connectivity. Functional connectivity looks at how brain activity is correlated in different brain regions (Biswal et al., 1995). Effective connectivity takes this one step further and aims to describe the direction of propagation of activity within functional networks (Friston et al., 2003; Park and Friston, 2013). The techniques used to study activity in the whole brain do not have the resolution to follow the propagation of neural activity between brain regions directly. Therefore, functional connectivity and effective connectivity are inferred from a statistical model applied to the data (Friston, 1994).

Learning modifies resting state functional connectivity (Lewis et al., 2009) and effective connectivity (Buchel et al., 1999; Fletcher et al., 1999). The changes in resting state networks may be rapid and last beyond the immediate post-training period. For instance, training on a visual perceptual learning task results in changes to resting state networks that can be seen at the end of a training session and persist for at least 24 h (Urner et al., 2013). Network reorganization has been followed over days in individuals learning a motor skill (Bassett et al., 2011, 2015). Brain activity was broken down into activity in sets of brain regions termed modules (Newman, 2006). The number of modules did not change during learning. In contrast, the brain regions in each module changed flexibly over days (Bassett et al., 2011).

These fMRI studies are based on spontaneous fluctuations in the blood-oxygen level dependent (BOLD) signal. Interpreting the findings in terms of neural connections is not easy for multiple reasons. Firstly, spontaneous fluctuations in the BOLD signal arise from a complex interplay between neural activity, anatomical connectivity and slow vascular fluctuations (Fox and Raichle, 2007; Leopold and Maier, 2012). Secondly, functional connectivity and anatomical connectivity overlap, but are not identical (Fox and Raichle, 2007). Thirdly, fMRI-based studies of connectivity face exactly the same problem that occurs with inferring connectivity from the firing of multiple neurons (Aertsen et al., 1989). It is not possible to distinguish whether the connectivity is due to physical connections between neurons or whether there are common or synchronous inputs that drive neural activity (Kelly and Garavan, 2005).

The rapid changes in connectivity seen with fMRI studies during a training session are unlikely to be due to rewiring as they occur too fast. Only, the long-lasting changes in functional or effective connectivity that persist over many days or weeks may have a component attributable to rewiring.

The contribution of rewiring has to be distinguished from functional changes in existing connections. These functional changes are not restricted to neuronal modifications, such as synaptic plasticity and altered neuronal excitability, but include the effects of glial cells (Sampaio-Baptista and Johansen-Berg, 2017). Oligodendrocytes, which myelinate axons (Tomassy et al., 2014), affect the speed of axonal conduction and, therefore, the flow of information through neural circuits. The speed of axonal conduction on the input pathways will modify the relative timing of input activity. A change in the relative timing of input activity affects how neurons integrate the different streams of input activity. Hence, changes in myelination may modify functional networks. This is supported by recent evidence suggesting that modifications to myelin thickness around axons can be driven by neural activity and support behavioural changes (Gibson et al., 2014; Mount and Monje, 2017).

In summary, functional networks reorganize over short and long time scales. Rewiring is unlikely to play a role in short-term reorganization, but may contribute to long-time scale reorganization. However, multiple other mechanisms can contribute. Consequently, dissecting out the precise contribution of rewiring to reorganization of functional networks requires that functional neuroimaging is combined with other cellular techniques. These experiments have yet to be done.

## 5 Effects of rewiring

There is good structural evidence that rewiring occurs in mammalian brains. What are the effects of rewiring? Rewiring reconfigures the structure of neural circuits. As a result, an immediate effect of rewiring is that it alters the neural activity within those circuits. However, the brain has multiple mechanisms for altering neural activity over the short and longer terms. What does rewiring add to existing mechanisms?

Neural activity can be altered over the short term by inhibition and by neuromodulatory inputs. Both inhibition and neuromodulators can change the way neural activity propagates through a static connectome (Bargmann and Marder, 2013). It has been proposed that neuromodulators dynamically control the strength of connections in prefrontal cortex, and that this is important for cognition (Arnsten et al., 2010). However, dynamic regulation of connection strength only works when connections are present. Hence, neuromodulatory control of connection strength is most effective in a densely-connected network. This is not the case for large parts of the nervous system. In particular, excitatory pyramidal neurons in the cortex tend to be sparsely connected to other pyramidal neurons (Barnes and Finnerty, 2010).

Mechanisms that alter neural activity over long time periods (days or longer) result in plasticity of the nervous system. Two broad classes of plasticity mechanism, changes in neuronal and synaptic plasticity, have been studied intensively (Barnes and Finnerty, 2010). Rewiring is a third class of plasticity mechanism, which has attracted less attention.

What can rewiring achieve above and beyond the other plasticity mechanisms? Rewiring connections consumes a great deal more resources than other plasticity mechanisms. What, then, might be the benefits of rewiring for learning? A fundamental problem for learning is that the architecture of the existing neural circuitry in the brain constrains learning; changes in synaptic strength and excitability can only be performed on existing connections between neurons. As a result, certain patterns of neural activity are easier to learn than others (Sadtler et al., 2014). This is supported by findings at the behavioural level, showing that information is learnt faster when it is consistent with prior knowledge and can be incorporated into existing schemas (Bartlett, 1932; Brod et al., 2016; Ranganathan et al., 2014; Tse et al., 2007).

In contrast, rewiring modifies the architecture of neural circuitry. This breaks the constraints imposed by the existing structure of a neural circuit on circuit output. The ability to modify the architecture of neural circuits may be particularly useful when new learning is not consistent with prior knowledge and does not fit into existing schema. As such, one possible advantage of rewiring is that it may enable greater reconfiguration of neural circuits and expand the capacity for learning. This is particularly beneficial for neural systems that are wired sparsely. This is the case for excitatory pyramidal neurons in the mammalian neocortex, which have low connectivity between neighbouring pyramidal neurons.

In conclusion, rewiring is a plasticity mechanism that enables neural activity to be changed over days. A major benefit of rewiring is that it offers a mechanism to escape the constraints imposed by existing brain circuitry.

## 6 Rewiring as a mechanism for learning

The experimental evidence suggests that the connectome can be rewired in the healthy brain during learning and experience-dependent plasticity. Does this mean that rewiring is a general mechanism for learning? To make this claim, a number of criteria need to be met. If rewiring is a general learning strategy, it should be present across a variety of learning paradigms. A strong case would be made if rewiring was necessary and sufficient for some forms of learning.

### 6.1 Does rewiring contribute to multiple forms of learning?

The relationship between rewiring the micro-connectome and learning has been probed with multiple paradigms involving repeated training (Table 1). For example, motor learning increases turnover of dendritic spines and results in the formation of new persistent dendritic spines (Xu et al., 2009; Yang et al., 2009). Improvement in behavioural performance correlates with the number of new persistent spines formed soon after training starts (Xu et al., 2009; Yang et al., 2009). This suggests that the early phases of learning are closely related to the formation of new dendritic spines (Xu et al., 2009; Yang et al., 2009). Once a task has been learnt, more training does not lead to further synaptogenesis. However, if a new task is learnt, then more dendritic spines are formed (Xu et al., 2009; Yang et al., 2009). This finding indicates that new synapses formed during learning are specific to the learning. Hence, there is good evidence that improvements in behavioural performance in tasks involving repeated training are related to the generation of new synapses.

In contrast, there is little information on whether rewiring contributes to one-trial learning. A major issue is identifying where the rewiring may occur and the extent of that rewiring. The brevity of one-trial learning suggests that if rewiring were involved, then it would most likely contribute to the consolidation phase of learning.

In summary, there is evidence that rewiring contributes to some forms of learning. Most studies have concentrated on learning over multiple trials, which are spread out over days. These studies indicate that learning is accompanied by rewiring of the micro-connectome and meso-connectome. Less is known about the contribution of rewiring to one-trial learning.

### 6.2 Is rewiring necessary and sufficient for learning?

The experimental data indicates that rewiring occurs during learning. However, to show that rewiring is a mechanism for learning, a causal relationship needs to be established. Ideally, this means proving that rewiring is necessary and sufficient for learning. Addressing the former requires demonstrating that preventing rewiring impairs learning. Addressing the latter requires showing that rewiring is sufficient to evoke learning. In practice, this remains a challenge.

Our understanding of the mechanisms involved in rewiring entire connections is limited (Table 2) (Barnes et al., 2015). The key problem is that the different plasticity mechanisms are unlikely to operate independently during learning. In fact, rewiring and synaptic plasticity mechanisms probably have to co-exist. This arises because the strength of new connections needs to be adjusted as the new connections are integrated into established neural circuits, as occurs for adult-born neurons (Bergami et al., 2015).

**Table 2:** Outstanding questions.

1)	*Does rewiring contribute to changes in neural firing in the brain?*
	This could be addressed by studying the firing of neurons longitudinally within a neural circuit in combination with following the structure of the connections between the neurons in the neural circuit over the same time period.
2)	*Is rewiring necessary or sufficient for any form of learning?*
	Experiments to address this could involve studying the consequences for learning when rewiring is prevented or induced.
3)	*How do connections rewire?*
	We know a great deal about how new connections are formed (Albieri et al., 2015; Knott et al., 2006). We know far less about how the loss of entire connections is co-ordinated (Barnes et al., 2015).
4)	*Can rewiring be harnessed to treat neurological and psychiatric disorders?*
	Further work needs to be done to establish the role of abnormal brain wiring in psychiatric and neurological diseases. The mechanisms that regulate rewiring will need to be understood if rewiring-based therapeutic interventions are to be developed and the risk of side effects mitigated.

The strongest evidence for a central role for rewiring in some forms of learning and memory comes from studies of adult-born neurons. Specifically, adult neurogenesis has been implicated in hippocampal-dependent memory formation in multiple ways (Anacker and Hen, 2017; Nakashiba et al., 2012; Sahay et al., 2011; Shors et al., 2001). Firstly, neurogenesis-induced rewiring in the hippocampus promotes memory transfer from the hippocampus to the neocortex (Kitamura et al., 2009). Secondly, neurogenesis can contribute to adaptive memory destabilisation, which facilitates the updating of memories during memory reconsolidation (Nader et al., 2000). Thirdly, neurogenesis may mediate adaptive forgetting. This is supported by a study showing that neurogenesis enhances forgetting of contextual fear memories in mice (Akers et al., 2014).

It has been more difficult to test whether rewiring is necessary or sufficient for learning that does not involve neurogenesis. Training mice on the rotarod results in increased formation of new dendritic spines that persist in the hindlimb motor cortex. Both the formation of new persistent spines and the improvement in behavioural performance are markedly reduced by a brief period of sleep deprivation immediately after training (Yang et al., 2014). This finding is consistent with the idea that the formation of new persistent dendritic spines contributes to learning. However, sleep has multiple effects on synapses, including homeostatic downscaling (de Vivo et al., 2017; Diering et al., 2017). Hence, the effects of sleep deprivation on dendritic spines does not provide definitive evidence on whether rewiring is either necessary or sufficient for learning.

In summary, studies involving adult-born neurons supply the clearest evidence that rewiring plays a central role in learning and memory. It has been harder to probe the role of rewiring in neural circuits without neurogenesis. A key issue is the lack of experimental manipulations that can tease apart the contribution of rewiring from other plasticity mechanisms. New approaches will be needed to address this issue and to advance our understanding of the mechanistic basis for learning.

## 7 Implications for neuropsychiatric diseases

It has been proposed that miswiring of the connectome is responsible for abnormal behaviour in certain psychiatric and neurological conditions (Di Martino et al., 2014). For example, it has been hypothesized that neurodevelopmental disorders, such as autism (Belmonte et al., 2004; Courchesne and Pierce, 2005) and schizophrenia (Stephan et al., 2006) are due to aberrant brain wiring. This idea has received some support. A combination of neuroimaging and neuropathological studies has suggested that both the structural and functional macro-connectome is modified. The brains of individuals with autism show a non-standard growth pattern with overgrowth during infancy followed by a decline in brain volume (Courchesne et al., 2011). In addition, the white matter tracts in the brains of individuals with autism are abnormal (Ecker et al., 2015; Herbert et al., 2004; Zikopoulos and Barbas, 2010). In parallel, studies of functional connectivity have found changes in multiple resting state brain networks (Anderson et al., 2011; Assaf et al., 2010; Gotts et al., 2012; Just et al., 2004; Kleinhans et al., 2008; Villalobos et al., 2005). At the meso- scale, patchy disorganization and cortical dyslamination in the neocortex of individuals with autism suggests that local brain circuits are abnormal (Stoner et al., 2014). Studies of the micro-connectome have tended to focus on the density of dendritic spines as a surrogate marker for synapse number. Spine density has been reported to be increased in the neocortex of individuals with autism (Hutsler and Zhang, 2010) and Fragile X syndrome (Irwin et al., 2000). In contrast, a reduction in dendritic spines has been observed in layer 2/3 prefrontal cortex in schizophrenia (Garey et al., 1998; Glantz and Lewis, 2000).

Whilst these studies suggest that there are abnormalities in the connectome of individuals with neuropsychiatric disorders (Di Martino et al., 2014), interpreting this evidence in terms of abnormal wiring is not straightforward. Histological studies of dendritic spines have two immediate issues. Firstly, they do not reveal whether synapse number has changed at existing connections. Secondly, they do not show the origin of the presynaptic neuron. Hence, they can’t distinguish between connections originating from different cell types and different brain regions. Current ‘macroscale’ techniques also possess limited ability to identify whether there is aberrant wiring in the brain. Changes in functional connectivity do not necessarily entail structural abnormalities in wiring (see Functional connectomics section). It will be important to clarify whether neuropsychiatric conditions are, at base, disorders of brain wiring because this would affect whether it would be worthwhile pursuing rewiring-based treatments (Table 2).

## 8 Harnessing rewiring therapeutically

One strategy to treat neurological and psychiatric diseases is to manipulate rewiring in the nervous system. The rewiring manipulations are combined with rehabilitation treatments to maximize symptom improvement. Research into the therapeutic applications of rewiring has tended to focus on conditions that damage the nervous system acutely. The central nervous system responds to acute damage by axonal sprouting and dendritic growth, which are the cellular basis for the rewiring that contributes to recovery (Carmichael et al., 2001; Dancause et al., 2005; Nudo et al., 1996; Silasi and Murphy, 2014).

The therapeutic goal in conditions with acute brain damage is either to boost the extent of rewiring or to accelerate rewiring to promote recovery. However, treatments that increase rewiring come with risks. Rewiring may disrupt established learning and memory and has the potential to cause disease (Dudek and Spitz, 1997). Therefore, enhancing rewiring will only offer substantial therapeutic benefits when the rewiring can be controlled (Benowitz and Carmichael, 2010).

One way to boost rewiring is to remove the molecular brakes that regulate rewiring. There are three classes of molecules that inhibit neuronal growth in the nervous system: myelin-associated proteins, such as Nogo A and myelin associated glycoprotein; extracellular matrix proteins, such as chondroitin sulphate proteoglycan; and axon guidance molecules, for example, ephrins, semaphorins, and netrins (Overman and Carmichael, 2014).

Agents that relieve the molecular brakes on rewiring have been tested in clinical trials, but have not shown dramatic efficacy to date. For example, a monoclonal antibody to myelin associated glycoprotein, which was given intravenously, has been used to treat stroke patients (Cramer et al., 2017). The study showed no benefits. However, it was not possible to distinguish whether the monoclonal antibody was ineffective or whether it failed to cross the blood brain barrier and reach its target.

Stimulation of a brain region has been used to promote rewiring. Rodent models of stroke enable detailed analyses of the cellular changes. Following a focal stroke in somatosensory cortex, optogenetic stimulation of thalamocortical afferents in the peri-infarct region boosts recovery from the stroke (Tennant et al., 2017). *in vivo* imaging showed an increase the number of axonal boutons on the terminal branches of thalamocortical axons. Large scale changes in the axonal arbor were not seen. The findings suggest that rewiring of the micro- and meso-connectomes has significant therapeutic effects.

Stem cell transplantation has been used to treat focal brain damage. The transplanted cells have multiple effects, but there is evidence that they promote structural changes in the brain (Andres et al., 2011). However, their main role in treatment is for neuronal replacement, which may involve rewiring and integration into brain circuitry to obtain maximal benefit.

## 9 Conclusion

There is growing evidence that the micro-connectome and meso-connectome rewire during experience-dependent plasticity and learning. Long-range connections between brain regions are unlikely to undergo extensive rewiring in the healthy adult brain. Instead, rewiring of long-range connections is limited to the terminal arbours of the projecting axons.

Although there is good evidence that the brain rewires, our understanding of the effects of structural rewiring on brain function lags far behind (Table 2, Q1). Brain plasticity is mediated by changes in synaptic strength and alterations to neuronal excitability, as well as rewiring. The contributions of the different types of plasticity mechanism need to be disentangled if we are to understand how they contribute to learning (Table 2, Q2). This work will help to elucidate how changes in behaviour emerge from modifications to brain circuitry.

Rewiring is a particularly valuable plasticity mechanism for brain regions where the probability of finding a connection between neurons is low, such as excitatory connections between pyramidal neurons in the neocortex. In brain regions with sparse connections, rewiring increases the number of ways that neural circuits can be reconfigured. This expands the capacity of the brain to learn. However, further work is needed to determine how formation and loss of entire connections between neurons is co-ordinated and regulated (Table 2, Q3). This knowledge is vital if we are to manipulate rewiring.

A deeper understanding of the effects of rewiring and how it is regulated would enable rewiring to be exploited therapeutically. Currently, treatments based on boosting rewiring are being developed for acute brain damage and for spinal cord repair. There is promise that rewiring based therapies could be used for neuropsychiatric disorders characterised by more widespread brain wiring abnormalities (Table 2, Q4). However, we will first need to understand better how to control rewiring to minimize the risk of making conditions worse because of aberrant rewiring.

## Conflicts of interest

None.
